# Targeted Next-Generation Sequencing Reveals Mutations in Non-coding Regions and Potential Regulatory Sequences of Calpain-3 Gene in Polish Limb–Girdle Muscular Dystrophy Patients

**DOI:** 10.3389/fnins.2021.692482

**Published:** 2021-10-14

**Authors:** Anna Macias, Jakub Piotr Fichna, Malgorzata Topolewska, Maria J. Rȩdowicz, Anna M. Kaminska, Anna Kostera-Pruszczyk

**Affiliations:** ^1^Department of Neurology, Medical University of Warsaw, Warsaw, Poland; ^2^Laboratory of Neurogenetics, Department of Neurodegenerative Disorders, Mossakowski Medical Research Institute, Polish Academy of Sciences, Warsaw, Poland; ^3^Laboratory of Molecular Basis of Cell Motility, Nencki Institute of Experimental Biology, Polish Academy of Sciences, Warsaw, Poland

**Keywords:** LGMD, *CAPN3*, non-coding, enhancer, silencer, calpain-3, LGMD2A, LGMDR1

## Abstract

Limb–girdle muscular dystrophy type R1 (LGMDR1) is caused by mutations in *CAPN3* and is the most common type of recessive LGMD. Even with the use of whole-exome sequencing (WES), only one mutant allele of *CAPN3* is found in a significant number of LGMDR patients. This points to a role of non-coding, intronic or regulatory, sequence variants in the disease pathogenesis. Targeted sequencing of the whole *CAPN3* gene including not only intronic, 3′ and 5′ UTRs but also potential regulatory regions was performed in 27 patients suspected with LGMDR1. This group included 13 patients with only one mutated *CAPN3* allele detected previously with exome sequencing. A second rare variant in the non-coding part of *CAPN3* was found in 11 of 13 patients with previously identified single mutation. Intronic mutations were found in 10 cases, with c.1746-20C>G variant present in seven patients. In addition, a large deletion of exons 2–8 was found in one patient. In the patients with no causative mutation previously found, we detected rare *CAPN3* variants in 5 out of 10 patients and in two of them in a compound heterozygous state. Rare variants within putative regulatory sequences distant from the *CAPN3* gene were found in 15 patients, although in 11 of these cases, other variants are deemed causative. The results indicate that intronic mutations are common in Polish LGMDR patients, and testing for non-coding mutations in *CAPN3* should be performed in apparently single heterozygous patients.

## Introduction

Limb–girdle muscular dystrophies (LGMD) are a group of hereditary progressive diseases of the skeletal muscle. Their genetic background is heterogeneous with about 30 different loci known to cause the disease. While LGMDs are rare diseases, with an estimated morbidity about 0.2–4 per 100,000 individuals (van der Kooi et al., 1996; Norwood et al., 2009), autosomal recessive LGMDR1 (in previous classification LGMD2A), caused by mutations in *CAPN3*, is one of the most frequent among them. In many European countries, including Poland, it is the most common form of an autosomal recessive LGMD (van der Kooi et al., 1996; Moore et al., 2006; Fanin et al., 2009; Fichna et al., 2018).

The phenotype of LGMDR1 can be variable. Most commonly, the disease manifests for the first time in the second decade of life, with progressive motor problems—usually, difficulty with stair climbing or standing up from a sitting position. In some cases, the shoulder girdle can be more affected than the pelvic girdle (Erb’s phenotype). Toe walking and contractures in lower limb muscles are common. Creatine kinase is typically high, decreasing with the progression of the disease. Skeletal muscle atrophy can be evident; however, hypertrophy of muscles is sometimes seen. Some cases have onset in the third decade of life or even later. The disease usually leads to significant disability, and loss of ambulation after 10–20 years is common.

Limb–girdle muscular dystrophy type R1 is caused by mutations in the *CAPN3* encoding calpain-3. It is a muscle-specific enzyme, belonging to the family of intracellular Ca^2+^-dependent cysteine proteases, the function of which is not fully explained. It acts as a sensor of muscle stress during muscle growth and remodeling after atrophy and exercise. It has also been suggested to play a role in the muscle intracellular Ca^2+^ transfer system (Kramerova et al., 2007; DiFranco et al., 2016).

The advent of high-throughput next-generation sequencing (NGS) methods has made the genetic diagnosis of LGMD much easier. Given that the phenotype of slowly progressing pelvic and shoulder girdle muscle weakness can have a very heterogeneous genetic background, the possibility to simultaneously identify rare variants in numerous genes is especially useful. Despite the recent progress in molecular diagnostic methods, still for up to half of LGMD cases, the causal mutation remains unknown (Ghaoui et al., 2015; Kuhn et al., 2016; Reddy et al., 2017; Topf et al., 2020). Excluding the oligogenic explanation, this can be either due to the causative mutations located in genes currently not linked with the disease or the presence of mutations in the already known LGMD genes that remain undetected. The latter can be the result of preferential use of whole-exome sequencing (WES) rather than whole-genome sequencing (WGS). WES is considered to be more cost effective, less time consuming, and the results are easier to interpret than WGS. However, by its very nature, WES cannot detect genomic variation outside the exome, i.e., in non-coding parts of the genome.

In a significant fraction (up to 20%) of patients with LGMDR1 phenotype, only one mutated allele of *CAPN3* is found upon sequencing of exomic DNA (Fanin et al., 2004; Fanin and Angelini, 2015; Topf et al., 2020). Apart from the known possibility of dominant inheritance pattern (Vissing et al., 2016, 2020; Martinez-Thompson et al., 2018; Cerino et al., 2020a; Gonzalez-Mera et al., 2021), this can be explained by the mutation of the other allele being localized in the non-coding regions that are not sequenced routinely. Intronic variants, including, but not limited to, splice site mutations, are found in LGMDR1 cases (Krahn et al., 2007; Fanin et al., 2012; Salem et al., 2012; Al-Harbi et al., 2016; Hu et al., 2019; Mavillard et al., 2019; Topf et al., 2020). Additionally, variants in the UTR, promoter, and regulatory regions not necessarily located within the *CAPN3* gene itself could also be pathogenic, although the role of potential regulatory DNA elements is not elucidated in LGMDs. Finally, a possibility of oligogenic inheritance in some LGMD cases cannot be excluded. A digenic mechanism has already been described in several neuromuscular disorders, including facioscapulohumeral dystrophy (Lemmers et al., 2012), a congenital myasthenic syndrome (Lam et al., 2017) and, recently, also in calpainopathy (Peddareddygari et al., 2018).

In a previously described cohort of 72 Polish LGMD patients (Fichna et al., 2018), WES identified 12 patients carrying only one mutated allele of *CAPN3*. In 10 of them, no other causal mutations were found in LGMD-related genes. The pedigree of these patients clearly indicated autosomal recessive mode of inheritance.

The aim of this study was to search for mutations in non-coding regions or potential regulatory sequences of *CAPN3* gene in patients carrying only one previously detected heterozygous *CAPN3* mutation or in patients with the LGMDR1 phenotype but negative results of WES.

## Materials and Methods

### Study Group

#### General Inclusion Criteria

The study group consisted of 27 non-consanguineous LGMD patients from a single neuromuscular center, in whom previously performed genetic tests had revealed only one heterozygous *CAPN3* mutation or had been negative for *CAPN3* and other causal mutations. The majority of the probands (*n* = 22) were selected from the cohort of 72 LGMD patients whose WES results were described previously (Fichna et al., 2018). The range of age at the LGMD diagnosis was 8–76 years, male-to-female ratio 10:17. The pedigree was indicative of autosomal recessive inheritance in 13/27 cases; one patient had second-degree relatives with muscular weakness, and the remaining 13/27 cases were sporadic. Basic clinical and demographic data are shown in Table 1.

**TABLE 1:** Demographic, basic clinical data, and previously identified *CAPN3* mutations in the study group patients.

Subgroup	No	Age at diagnosis; gender	Pedigree	Age at onset of overt clinical symptoms	Brief clinical summary	Previously identified *CAPN3* mutation	Putative other causal gene mutations
A WES-CAPN3het	A1	10 F	AR	Early childhood	Severe phenotype, loss of ambulation in first decade	p.Glu107Lys	*FKRP*: p.Leu93Pro, p.Arg270Cys
	A2	76 M	AR	Adolescence	Ambulation preserved into 8th decade	p.Glu217Lys	*LMNA*: p.Gly523Arg
	A3	56 F	AR	Adulthood	Ambulation with assistance	p.Gly161Arg	
	A4	57 F	AR	Adolescence	Ambulation with assistance	p.Gly234Arg	
	A5	41 F	AR	Adulthood	Progressive ambulation difficulties; waddling gait	c.598-612 delGTTCTGGAGTGCTCT	
	A6	35 M	AR	Adulthood	Asymmetrical upper limb onset; ambulation preserved	c.598-612 delGTTCTGGAGTGCTCT	
	A7	42 M	AR	Adulthood	Ambulation preserved	c.598-612 delGTTCTGGAGTGCTCT	
	A8	44 M	AR	Adulthood	Upper limb predominant; ambulation preserved	c.598-612 delGTTCTGGAGTGCTCT	
	A9	16 F	AR	Adolescence	Progressive ambulation difficulties	p.Thr706Arg	
	A10	56 F	AR	Adulthood	Progressive limb–girdle weakness; ambulation preserved	p.Arg533Ser	
	A11	15 M	AR	Adolescence	Progressive ambulation difficulties	c.550delA	
	A12	31 F	AR	Adolescence	Progressive ambulation difficulties; ambulatory with effort	c.1722delC	
	A13	30 F	Sporadic	Adulthood	Myalgia and minimal weakness	p.Asp753Asn	
B WES-neg	B1	14 F	Sporadic	Early childhood	Toe walking in childhood; ambulation preserved	–	
	B2	48 F	Maternal aunt and cousin affected	Childhood	Slowly progressive; ambulation preserved	–	*CCDC78*: p.Arg103Gln
	B3	10 F	AR	Childhood	Quickly progressing; waddling gait	–	
	B4	8 F	Sporadic	–	Asymptomatic hyper-CKmia	–	
	B5	36 F	Sporadic	Adolescence	Slowly progressing; ambulation preserved	–	
	B6	17 M	Sporadic	Adolescence	Ankle contractures; ambulation Preserved	–	
	B7	34 M	Sporadic	Early adulthood	Miyoshi myopathy phenotype	–	
	B8	9 F	Sporadic	–	Asymptomatic hyper-CKmia	–	
	B9	14 F	Sporadic	Childhood	Limb–girdle weakness, ambulation preserved	–	*COL6A3*: p.Arg2142Ter, p.Lys2483Glu
	B10	41 M	Sporadic	Adulthood	Progressive limb–girdle weakness; waddling gait	–	*COL6A3*: p.Glu1386Lys, p.Arg2420Trp *CACNA1S*: p.Thr349Ser
C Sang-CAPN3het	C1	27 F	Sporadic	Childhood	Progressive limb–girdle weakness; wheelchair bound	c.550delA	
	C2	12 F	Sporadic	Childhood	Progressive limb–girdle weakness; wheelchair bound in 3rd decade	c.550delA	
	C3	12 M	Sporadic	Childhood	Progressive limb–girdle weakness; ankle contractures; wheelchair bound in 3rd decade	c.550delA	
	C4	23 M	Sporadic	Childhood	Progressive limb–girdle weakness; ankle contractures; wheelchair bound in 3rd decade	c.550delA	

#### Subgroups

Three subgroups of the patients were defined for the study purpose.

Subgroup A (*n* = 13) comprised heterozygotes with one mutated *CAPN3* allele revealed either by WES or a targeted NGS panel of myopathy-related genes. The subgroup was dubbed “WES-CAPN3het.” Within this subgroup, 7/13 patients had a missense *CAPN3* point mutation (different for each patient), 4/13 patients had an in-frame c.598-612 deletion, one patient had the common c.del550A mutation, and one patient carried a c.1722delC deletion. In two patients, besides one *CAPN3* mutation, also other LGMD-related gene mutations (at the time considered causative) had been found (patient A1—*FKRP* mutation and patient A2—*LMNA* mutation).

Subgroup B (*n* = 10) included patients with neither *CAPN3* mutation nor other certainly causative pathogenic mutation found selected arbitrarily from the original cohort based on the phenotype concordant with LGMDR1. The subgroup was dubbed “WES-neg.” In the WES-neg group, three patients had mutations in other LGMD genes, which could be causative but were not fully concordant with the phenotypes of the patients (see Table 1). Other rare variants in LGMD-related genes, revealed by WES, were considered probably not causative and are shown in Supplementary Table 1.

Subgroup C (*n* = 4) comprised patients previously tested only by Sanger sequencing for the most common *CAPN3* mutations and found heterozygous for them (the subgroup was dubbed “Sang-CAPN3het”). All patients in this subgroup were heterozygous for c.del550A.

**FIGURE 1:**
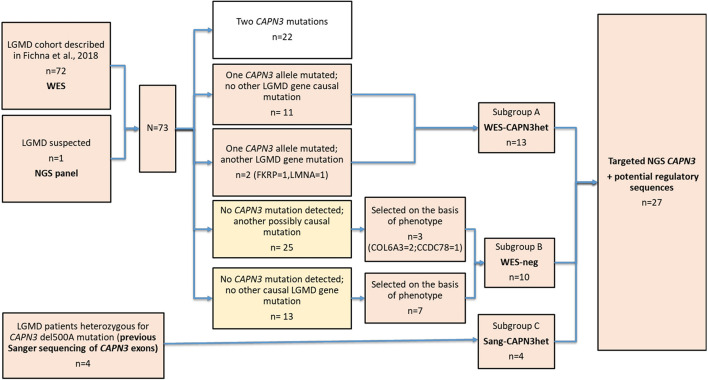
Flow chart of the study group selection and division into subgroups.

The patient selection algorithm is shown in Figure 1.

#### Clinical Criteria of Limb–Girdle Muscular Dystrophies

Limb–girdle muscular dystrophies was defined as progressive muscle weakness and atrophy of the pelvic and shoulder girdle muscles, as well as proximal limb muscles, without involvement of facial muscles. The diagnosis was made on the basis of clinical assessment and muscle biopsy. Childhood cases with early-onset asymptomatic persistent hyperCKemia were included when the muscle biopsy revealed features of muscular dystrophy. Miyoshi myopathy phenotypes (muscular dystrophy with predominant calf atrophy and high CK level) were also included. To exclude Becker muscular dystrophy cases, we included only these male patients who had either similarly affected female siblings or previously negative MLPA (multiplex ligation-dependent probe amplification) results (which excluded large deletions or duplications in the dystrophin gene), together with the normal muscle immunofluorescence staining for dystrophin. Patients with facioscapulohumeral muscular dystrophy, Emery–Dreifuss muscular dystrophy, and myotonic dystrophy types 1 and 2, and other myopathies (congenital, metabolic, mitochondrial, myofibrillar, and inflammatory) were excluded based on their clinical, electrophysiological, genetic testing, or histopathological characteristics, if appropriate.

Demographic and basic clinical data of the patients are presented in Table 1. More detailed clinical data are given in Supplementary Table 2.

### Ethics Approval and Consent to Participate

The study was approved by the Ethics Committee of the Warsaw Medical University (Warsaw, Poland) in compliance with national legislation and the Code of Ethical Principles for Medical Research Involving Human Subjects of the World Medical Association (approval number 163/2017). Written consent was obtained from all patients and healthy individuals according to the Declaration of Helsinki (BMJ 1991; 302:1194). The authors are very grateful to all the families for their participation in this study.

### Genetic Analysis

DNA was extracted from peripheral blood using standard methods. Targeted sequencing was performed commercially at Novogene (People’s Republic of China). Target sequences included the whole *CAPN3* gene flanked with 5 kbp and additionally putative *CAPN3* enhancers selected based on data from the GeneCards database (Supplementary Table 3). Selected DNA sequences were captured with Agilent’s SureSelectXT Target Enrichment probes. Created custom library was sequenced on an Illumina HiSeq platform with 150-bp paired-end reads. Fast read files were generated from the sequencing platform *via* the Illumina pipeline. Adapter sequences in the raw data were removed, and low-quality reads discarded. The obtained “clean” paired-end reads were aligned to the human reference genome GRCh38 using the Burrows–Wheeler Alignment (BWA) package. Duplicate reads were removed with Picard, and base quality Phred scores were recalibrated using GATK covariance recalibration. The obtained 2 giga-bases of aligned sequence data per sample resulted in 300× median coverage of the target capture regions with 100% of target bases covered at least 30×. The alignments were viewed with Integrative Genomics Viewer. SNVs (single-nucleotide variants) and indel (small insertion/deletion) variants were called using the GATK Unified Genotyper. Annovar was used for variant annotation.

The revealed variants were filtered using frequency data from the gnomAD database. Rare variants, defined as of minor allele frequency (MAF) <1% in the Non-Finnish European population (NFE) in gnomAD, were further analyzed in this paper. Additionally, low-frequency variants of MAF between 1 and 3% were listed and are also presented separately. Low-frequency variants of MAF between 3 and 5% were not included in the paper.

Initially, as in the earlier WES study, one proband was tested from each family. In cases with rare variants found, available family members were also tested to analyze the genotype–phenotype cosegregation.

### Western Blot

Expression of *CAPN3* protein in biopsied skeletal muscle, obtained either from biceps brachii or quadriceps, was assessed with Western blot (Towbin et al., 1979). Frozen skeletal muscle biopsy specimens were homogenized at 1:20 (w/v) ratio in phosphate-buffered saline (PBS) supplemented with protease inhibitors. The same volume of the low-speed supernatants was loaded per each well of 8 or 10% SDS-polyacrylamide gel. After the transfer onto the nitrocellulose membrane (see Supplementary Figure 1), calpain-3 was detected with the primary monoclonal antibody raised against amino acids 1–80 mapping at the N-terminus of calpain-3 of human origin (sc-365277, Santa Cruz Biotechnology, Inc., United States) followed by incubation with the secondary horseradish peroxidase conjugated with anti-mouse antibody and ECL (enhanced chemiluminescence) substrates (WBKL S0500, Millipore, United States). Biopsy specimens (biceps brachii) from non-LGMD patients were used as a control. Those were obtained from female patients (age 15–41) that turned to the clinics with myopathy symptoms, but their biopsies did not reveal any pathological changes (samples C1 and C3) or prediagnosed with sarcoidosis (C2) with very mild uncharacteristic changes in the muscle sample. For each sample, levels of GAPDH (glyceraldehyde 3-phosphate dehydrogenase), detected with monoclonal anti-GAPDH antibody (MAB374; Millipore, United States) or cytoskeletal β-actin isoform, detected with monoclonal anti-β-actin antibody (A5441; Sigma-Aldrich, United States) were also assessed and served as loading controls. β-actin detection was performed on the stripped membranes prior used for detection of GAPDH.

## Results

Targeted sequencing revealed 296 variants including 122 in the *CAPN3* gene and 174 in its putative regulatory regions or flanking sequences. Further filtering using frequency data from the gnomAD database reduced these numbers to, respectively, 25 and 17 rare (MAF < 1% in the NFE in gnomAD) variants. The present targeted sequencing confirmed the earlier WES results, detecting all previously found variants. However, one additional missense mutation, undetected with WES, was identified.

### Diagnostic Yield of the Whole *CAPN3* Gene Next-Generation Sequencing

In the whole study group, one or more rare variants in the whole *CAPN3* gene, not identified previously by WES or Sanger sequencing, were found in 22/27 patients.

In the WES-CAPN3het subgroup, a second mutation in the *CAPN3* gene was found in 12/13 patients, including also the probands carrying putative pathogenic mutations in other genes (probands A1 and A2).

In the WES-neg subgroup, compound heterozygous *CAPN3* mutations were found in 2/10 patients, and a single *CAPN3* mutation in the further 3/10.

In the Sang-CAPN3het subgroup, a second *CAPN3* mutation was found in all (4/4) patients.

We were unable to find any previously undetected rare *CAPN3* variant in 6 patients—1/13 from the WES-CAPN3het subgroup and 5/10 from the WES-neg subgroup.

Relaxing the frequency-filtering threshold to 3% minor allele frequency (MAF) in the NFE revealed three more variants in two patients (B5 and B8).

Detailed data are shown in Table 2.

**TABLE 2:** Rare variants in the *CAPN3* gene, in its putative regulatory sequences, and other possibly pathogenic variants in limb–girdle muscular dystrophy (LGMD)-related genes in the study group.

Patient no.	*CAPN3* mutations (reference sequence NM_000070.2)		Inheritance: maternal M, paternal P, not assessed n/a	Rare variants in potential regulatory regions (NFE frequency < 1%)	Other rare variants with NFE frequency 1–3%	Putative causal mutations in other LGMD genes
	Previously identified	Found by NGS *CAPN3* targeted sequencing				
A1	c.319G>A p.Glu107Lys	c.309+7931C>T	*n/a*	–	–	*FKRP*: p.Leu93Pro, p.Arg270Cys

A2	c.649G>A p.Glu217Lys	c.1746-20C>G	*n/a*	g.42406949C>G	g.42353858T>C	*LMNA*: p.Gly523Arg
		c.310-893C>T				
		c.1782+223A>G				

A3	c.481G>A p.Gly161Arg	c.1746-20C>G	*n/a*	g.42406949C>G	-	–
		c.310-893C>T				
		c.1782+223A>G				

A4	c.700G>A p.Gly234Arg	c.1746-20C>G	*n/a*	g.42341662C>G	g.42268797A>G	–
		c.310-893C>T		g.42406949C>G		
		c.1782+223A>G				

A5	c.598-612del p.Phe200_Leu204del		*M*			–
		c.1746-20C>G	*P*	g.42350187C>T		
		c.310-893C>T		g.42406949C>G		
		c.1782+223A>G				

A6	c.598-612del p.Phe200_Leu204del		*M*			–
		c.1746-20C>G	*P*	g.42347848C>T		
		c.310-893C>T		g.42406949C>G		
		c.1782+223A>G				

A7	c.598-612del p.Phe200_Leu204del	c.1746-20C>G	*n/a*	g.42406949C>G		–
		c.310-893C>T				
		c.1782+223A>G				

A8	c.598-612del p.Phe200_Leu204del	c.1746-20C>G	*n/a*	g.42341662C>G		–
		c.310-893C>T		g.42406949C>G		
		c.1782+223A>G				

A9	c.2117C>G p.Thr706Arg	c.309+4853C>G	*P*		g.42456527C>T	–
		c.1194-9A>G	*M*	g.42459106T>C	g.42359043C>G	

A10	c.1599G>C p.Arg533Ser	c.1355-158C>A	*n/a*	g.42268785G>A	g.42347798T>C	–

A11	c.550delA		*P*	–	–	–
		c.*32A>G	*M*			

A12	c.1722delC		*M*	–	–	–
		del exons 2-8 (g.42364582_42395592del)	*P*			

A13	c.2257G>A p.Asp753Asn	–	*P*	g.42354194C>T		–
		–	*M*		g.42350479A>G	

B1	–	c.1333G>A p.Gly445Arg	*M*			–
		c.1746-20C>G	*P*	g.42341662C>G		
		c.310-893C>T		g.42406949C>G		
		c.1782+223A>G				

B2	–	c.309+1668A>T	*P*			*CCDC78*: p.Arg103Gln
		c.309+5112C>T	*M*		g.42352416T>C	

B3	*–*	c.1746-20C>G	*n/a*	g.42406949C>G	g.42650498C>T	–
		c.310-893C>T				
		c.1782+223A>G				
	**Previously identified**	**Found by NGS *CAPN3* targeted sequencing**				

B4	–	c.310-8109A>G	*M*	g.42339146C>T	–	–
			*P*	g.42420121T>C		

B5	*–*	c.310-11129G>A	*n/a*	g.42013048G>A	c.1194-856T>C	–
				g.42448028G>A	c.1524+81C>T	
					g.42454737A>G	

B6	–	–	*n/a*	–	g.42342695G>A	–
					g.42347798T>C	

B7	–	–	*n/a*	-	-	-

B8	–	–	*n/a*		g.42011991G>T	-
					c.1354+543C>T	

B9	–	–	*n/a*	g.42011745T>C	g.42341108C>T	*COL6A3*: p.Arg2142Ter, p.Lys2483Glu
					g.42650498C>T	

B10	–	–	*n/a*	–	–	*COL6A3*: p.Glu1386Lys, p.Arg2420Trp
						*CACNA1S*: p.Thr349Ser

C1	c.550delA	del exons 2-8 (g.42364582_42395592del)	*n/a*	–	–	n/a

C2	c.550delA	c.759_761delGAA p.Lys254del	*n/a*	–	g.42286663T>C	n/a
					g.42341108C>T	

C3	c.550delA	c.550delA	*n/a*	–	–	n/a

C4	c.550delA	c.598-612del p.Phe200_Leu204del	*n/a*	–	–	n/a

### Rare *CAPN3* Variants Found

Previously undetected coding sequence mutations were found in one WES-CAPN3het, one WES-neg, and all four Sang-CAPN3het patients. These included:

- a missense point mutation in a WES-neg patient (B1),
- a large deletion comprising exons 2–8 in one WES-CAPN3het proband and one Sang-CAPN3het proband,
- an inframe c.759_761delGAA deletion, an inframe c.598_612 deletion, and a homozygous c.550delA—in the remaining three Sang-CAPN3het patients.

Rare variants in non-coding sequences were found in 10/13 WES-CAPN3het patients and 5/10 WES-neg patients. Among them, a haplogroup of three intronic variants: c.310-893C>T, c.1746-20C>G, and c.1782+223A>G was the most prevalent, found in 9/27 cases: seven of WES-CAPN3het patients and two of WES-neg patients. In four cases, it was present in combination with a missense *CAPN3* mutation (different in each patient) and in four other cases—with a c.598-612 deletion. Only in one proband were these three variants the sole rare *CAPN3* variants found.

One WES-CAPN3het proband carried a probable splice-site mutation c.1194-9A>G.

Different deep intronic *CAPN3* mutations were found in seven patients (3 WES-CAPN3het, 4 WES-neg), in one case in a compound heterozygous state (proband B2).

A mutation in the 3′UTR was revealed in one WES-CAPN3het proband.

### Rare Variants in Putative Regulatory Sequences

Variants in the putative regulatory sequences distant from the *CAPN3* gene were found in all three subgroups of probands. Fifteen out of 27 patients were carrying at least one rare (NFE MAF < 1%) variant in a putative regulatory sequence (10 WES-CAPN3het patients, 5 WES-neg patients). Relaxing the frequency-filtering threshold to NFE MAF <3% increased the number of variant carriers to 19/27 (10 WES-CAPN3het patients, 8 WES-neg patients, 1 Sang-CAPN3het patient), 15 of which were carrying more than one variant.

Twenty-six different variants in putative regulatory sequences distant from the *CAPN3* gene were found, 13 rare (NFE MAF < 1%) and 13 low-frequency ones (NFE MAF 1–3%). Among the rare variants, g.42406949C>G was present in nine patients and seems to be in linkage disequilibrium with the three intronic variants described above (c.310-893C>T, c.1746-20C>G, and c.1782+223A>G). Four other variants were present in more than one patient, among which only g.42341662C>G was identified rare in three cases.

Detailed data are shown in Table 2. For the whole list of rare non-coding and regulatory sequence variants found, see Table 3.

**TABLE 3:** Summary of all rare and low-frequency variants in *CAPN3* non-coding regions and potential regulatory regions (NFE frequency < 3%) found in 27 patients.

No.	Variant name	dbSNP	gnomAD allele frequency	gnomAD Non-Finnish European allele frequency	Highest population allele frequency (if Non-NFE>1%)	Number of occurrences in the study group	Patient(s) carrying the variant
1	g.42011745T>C	rs192168968	0.00465	0.00585	0.01215 Finnish	1	B9
2	g.42011991G>T	rs1530835	0.0862	0.01902	0.23115 East Asian	1	B8
3	g.42013048G>A	rs527967617	0.00207	0.00307		1	B5
4	g.42268785G>A	rs189821654	0.00042	0.00047		1	A10
5	g.42268797A>G	rs113607427	0.02049	0.02531	0.0518 Finnish	1	A4
6	g.42286663T>C	rs140014238	0.00739	0.01266		1	C2
7	g.42339146C>T	rs571784084	0.0001	0.00007		1	B4
8	g.42341108C>T	rs112234691	0.01669	0.01342	0.02893 Ashkenazi	2	B9, C2
9	g.42341662C>G	rs901445707	0.00026	0.0002		3	A4, A8, B1
10	g.42342695G>A	rs181857231	0.00863	0.01154	0.01866 Finnish	1	B6
11	g.42347798T>C	rs35022353	0.01036	0.01572	0.01747 Finnish	2	A10, B6
12	g.42347848C>T	.	.	.		1	A6
13	g.42350187C>T	rs1358635511	0.00052	0.00067		1	A5
14	g.42350479A>G	rs118166665	0.01054	0.01577		1	A13
15	g.42352416T>C	rs147930287	0.01052	0.01439	0.02148 American	1	B2
16	g.42353858T>C	rs112710998	0.0124	0.02072		1	A2
17	g.42354194C>T	rs148085740	0.00045	0.00073		1	A13
18	g.42359043C>G	rs140082921	0.01182	0.01667	0.02232 Finnish	1	A9
19	g.42361782A>T c.309+1668A>T	rs573055804	0.00304	0.005		1	B2
20	g.42364967C>G	.	.	.		1	A9
21	g.42365226C>T c.309+5112C>T	rs896433255	0.00013	0.0002		1	B2
22	g.42368045C>T c.309+7931C>T	rs1321681085	0.00003	0.00007	0.0189 Finnish	1	A1
23	g.42373354G>A c.310-11129G>A	rs28364380	0.00539	0.00646	0.03642 Ashkenazi	1	B5
24	g.42376374A>G c.310-8109A>G	rs952926569	0.0001	0.00007		1	B4
25	g.42383590C>T c.310-893C>T	rs546534693	0.00532	0.00694	0.01402 Finnish	9	A2, A3, A4, A5, A6, A7, A8, B1, B3
26	g.42398636T>C c.1194-856T>C	rs150891938	0.02096	0.02282	0.03876 Ashkenazi	1	B5
27	g.42399483A>G c.1194-9A>G	rs374665929	.	.		1	A9
28	g.42400195C>T c.1354+543C>T	rs28364475	0.06527	0.02506	0.16648 African	1	B8
29	g.42401483C>A c.1355-158C>A	rs551704617	0.00008	0.00008		1	A10
30	g.42401891C>T c.1524+81C>T	rs28364486	0.0094	0.01228	0.03974 Ashkenazi	1	B5
31	g.42403721C>G c.1746-20C>G	rs201892814	0.00546	0.007	0.01403 Finnish	9	A2, A3, A4, A5, A6, A7, A8, B1, B3
32	g.42404000A>G c.1782+223A>G	rs141693768	0.00546	0.007	0.01433 Finnish	9	A2, A3, A4, A5, A6, A7, A8, B1, B3
33	g.42406949C> G	rs146933502	0.00539	0.007	0.01431 Finnish	9	A2, A3, A4, A5, A6, A7, A8, B1, B3
34	g.42411805A>G c.*32A>G	.	.	.		1	A11
35	g.42420121T>C	.	.	.		1	B4
36	g.42448028G>A	rs200888168	0.00097	0.00127	0.01656 Ashkenazi	1	B5
37	g.42454737A>G	rs116853380	0.01896	0.02732	0.03231 Finnish	1	B5
38	g.42456527C>T	rs145558898	0.01701	0.02313	0.03129 Finnish	1	A9
39	g.42459106T>C	rs551802296	0.00226	0.00367		1	A9
40	g.42650498C>T	rs12324135	0.13987	0.0128	0.43277 African	2	B3, B9

### Segregation of the *CAPN3* and Non-coding Sequence Mutations in Families

**FIGURE 2:**
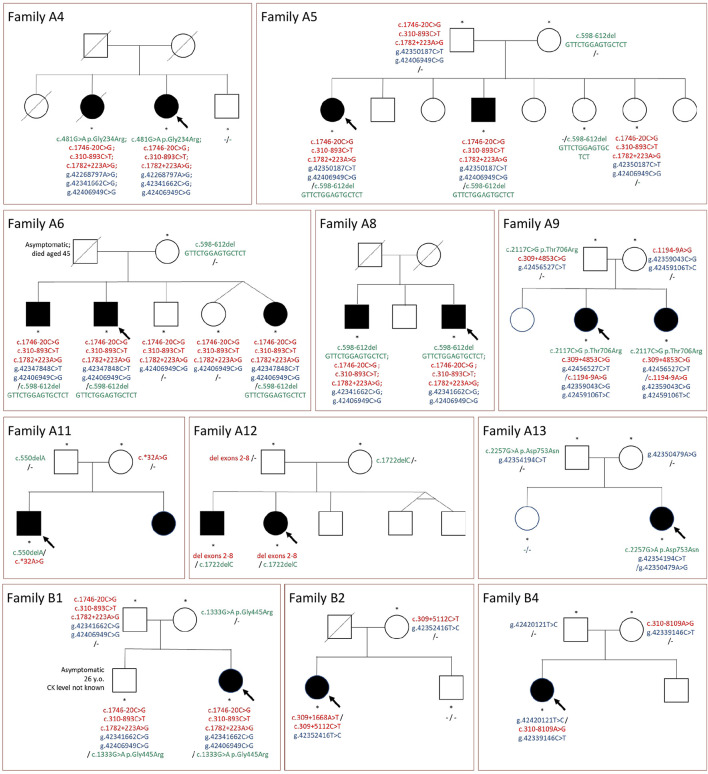
Pedigrees of the families and segregation of selected mutations. Green color, mutations in coding sequences; red color, mutations in non-coding regions or large deletions not detected with WES; blue color, rare variants in potentially regulatory sequences; “-”, no rare variant detected; arrow, indicates the proband; asterisks, tested family members.

Genetic testing of both parents could be performed in nine cases. For all these probands, we confirmed a compound heterozygous state for rare variants (including variants in potential regulatory regions). The linked variants described above (c.310-893C>T, c.1746-20C>G, c.1782+223A>G, and g.42406949C>G) were inherited together from one parent in all three families in which parents were tested (Table 2 and Figure 2).

Sibling testing was performed in nine families. The pedigrees are shown in Figure 2. All affected siblings tested shared the same *CAPN3* variants with the proband. Non-affected siblings mostly were carriers of one mutation or carried none of the variants. The only exception was an asymptomatic adult brother in family B1, sharing all the rare variants with the paucisymptomatic proband. This finding is further discussed below.

### *CAPN3* Expression in Biopsied Muscle

**FIGURE 3:**
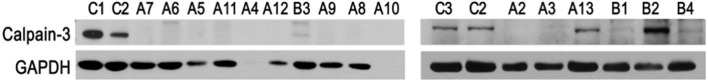
Analysis of *CAPN3* expression in the skeletal muscle by Western blotting. Samples C1–C3, specimens of non-LGMD controls; samples A2–A13, specimens of WES-CAPN3het patients and samples B1–B4, specimens of WES-neg patients. GAPDH and β-actin serve as internal loading control. C1–C3, biceps brachii; A2–A3, A5, A9–A12, and B2–B4, quadriceps; A4, A6–A8, and B1, biceps brachii.

To assess whether the mutations found herein could affect expression of *CAPN3*, the patients’ muscle biopsy specimens were subjected to the Western blot analysis aimed at assessment of calpain-3 (Figure 3).

In the WES-CAPN3het subgroup (patients A2–A13), calpain-3 (M.W. ∼80 kDa) was undetectable in 11/12 samples (patients A2–A12) and only in patient A13 was calpain level comparable with controls. Muscle biopsy specimen of patient A1 was not available.

In WES-neg patients (patients B1–B4), calpain-3 was barely detectable in patient B1, and only a trace of the protein was found in patients B3 and B4. However, the amount significantly higher than in the control was detected in patient B2, despite the fact that the overall level of the protein was comparable to that of the sample of patient B1, where calpain-3 was barely detectable (Supplementary Figure 1). For the remaining patients, Western blotting was not performed as neither *CAPN3* mutation was found nor biopsy specimen was available.

### Retrospective Assessment of the Original Limb–Girdle Muscular Dystrophy Cohort for Presence of Variant c.1746-20C>G

The c.1746-20C>G variant was not automatically reported in the original WES results of the 72 LGMD patients (Fichna et al., 2018); however, due to its location in the flanking region, it was possible to reassess the data using modified filtering criteria. We retrospectively verified the presence of the c1746-20C>G variant in the remaining 50 patients from the original LGMD cohort (Fichna et al., 2018), finding it in 2/50 cases. Both probands were originally diagnosed with calpainopathy, each of them carrying two known pathogenic *CAPN3* mutations in the coding sequence. Segregation of variants within families was not determined in these cases. The prevalence of the c.1746-20C>G variant in the original cohort is presented in Table 4.

**TABLE 4:** Prevalence of the c.1746-20C>G variant in the original cohort of 72 patients (Fichna et al., 2018).

Probands in the original cohort		Number of patients		
of 72 LGMD patients		Number of patients		
		**With c.1746-20C**>**G variant**	**Without c.1746-20C**>**G variant**	**Total**
	
Exonic mutations found	Two heterozygous exonic *CAPN3* mutations or one homozygous exonic *CAPN3* mutation	2	20	22
	One heterozygous exonic *CAPN3* mutation	8	5	13
	Causative mutations in other LGMD-related genes only	0	25	25
	No causative mutation in LGMD-related genes	1	11	12
Whole cohort		11	61	72

### Genotype–Phenotype Correlations

Four unrelated patients had the c.598-612del/c.1746-20C>G genotype (patients A5–A8). They all presented uniformly with mature adult disease onset and no contractures. In 2/4 cases, upper-limb weakness was predominant (Erb phenotype).

Four other patients with the c.1746-20C>G variant (and three variants in linkage disequilibrium) in combination with missense *CAPN3* mutations presented various time of onset; however, weakness progression was relatively slow, and all four patients (A2–A4 and B1) were ambulatory (sometimes with assistance), and in the case of patient A2 even until the eighth decade of life. None of these patients presented with permanent contractures. Patient B1 was toe walking during childhood; however, contractures resolved in adult life, and on follow-up the patient exhibited no motor deficit at the age of 26.

The only patient with c.1746-20C>G without a mutation within the *CAPN3* coding region (patient B3) was, in contrast, severely affected. The patient also carried an additional rare variant in a potential regulatory region, but it is not known whether it was on the same or different chromosome.

The classical phenotype with adolescent onset, contractures, and early ambulation loss was characteristic for patients with the most common European del550A mutation in combination with other coding sequence mutations (patients C1–C4).

Patients with deep intronic mutations other than the trio in linkage disequilibrium presented with variable disease onset and progression rate (A1, A10, B2, B4, B5); however, except for patient A1 (who also had pathogenic variants in *FKRP*), the clinical course tended to be milder than in subgroup C.

In two patients (A13 and B4), coding sequence or intronic mutation was found only on one allele, and the other allele carries a rare variant in potential regulatory sequence. Both these patients presented with very mild symptoms, with the caveat that their observation time was rather short.

Detailed clinical data for all 27 patients are presented in Supplementary Table 1.

### Reassessment of Diagnosis

For patient A1, who had originally been diagnosed with LGMDR9 (2I) (*FKRP* mutations), and presented with a severe, Duchenne-like phenotype, the LGMDR9 diagnosis was still considered most plausible, despite the presence of *CAPN3* rare variants.

For patient A2, a *LMNA* mutation had been previously suspected to be causative, but autosomal recessive inheritance indicated another genetic background. LGMDR1 was diagnosed after targeted NGS of *CAPN3*.

For patients A3–A12, the original diagnosis of LGMDR1 was confirmed, based on the genetic results, clinical phenotype, and Western blot.

For patient A13, LGMDR1 cannot be proven, although this diagnosis is clinically probable.

Among the WES-negative patients, only patient B1 was diagnosed with LGMDR1. In the remaining patients, the genetic background of LGMD is still not solved.

For all four Sang-CAPN3het patients, the diagnosis of LGMDR1 was confirmed.

## Discussion

In a significant fraction of LGMDR patients, only one mutant allele of the calpain-3 gene has been reported (Fanin et al., 2004; Fanin and Angelini, 2015; Fichna et al., 2018; Topf et al., 2020), which cannot be reconciled with the well-established autosomal recessive mode of inheritance. We hypothesized that this apparent discrepancy could be explained by the presence of *CAPN3* variants located in regions overlooked in a typical genetic analysis, even using WES technique. Such variants could be important in the disease pathogenesis or might at least modulate clinical phenotype. To clarify this, we have performed a comprehensive analysis of the whole *CAPN3* locus in 27 patients with a clinical diagnosis of LGMD.

Targeted sequencing revealed a second rare variant in the non-coding regions of *CAPN3* in 11/13 patients from the original cohort (Fichna et al., 2018) with a previously identified single-coding sequence mutation. Intronic mutations were most frequent (10/13), with the c.1746-20C>G variant most prevalent, found in seven patients. In addition, a large deletion of exons 2–8 was found in one patient. In patients without any causative mutation previously found, we detected rare *CAPN3* variants in 5/10 patients, in two of them in a compound heterozygous state. A detailed discussion of the possible phenotypic significance of the identified variants is provided below.

### Pathogenicity of the Identified *CAPN3* Variants

In 10 families, the co-segregation with the disease symptoms strongly points to the causative role of the *CAPN3* non-coding variants (Figure 2). The only exception (family B5) is discussed further below. In the remaining cases, there were no family members available; therefore, the pathogenicity of the identified variants could not be confirmed. Of note, in the study group, rare *CAPN3* non-coding variants were frequently accompanied by rare variants in putative regulatory sequences; therefore, the hypothetical permissive role of the latter should not be utterly dismissed.

Reduction of calpain-3 protein amount in most Western blot samples further supports the pathogenicity of the identified variants, as it is highly specific for a primary calpainopathy (Fanin and Angelini, 2015). Apart from LGMDR1, loss of *CAPN3* protein was unambiguously demonstrated only in titinopathy (LGMDR10) (Haravuori et al., 2001; De Cid et al., 2015). In the group presented here, no known pathogenic *TTN* variants were found. Heterozygous rare *TTN* variants of unknown significance (VUS) were common (Supplementary Table 1), but in light of their high prevalence in other LGMD patients (Fichna et al., 2018), as well as in general population (Akinrinade et al., 2015), their causative role is much more disputable than that of *CAPN3* variants found.

### Variant c.1746-20C>G

The most common mutation not revealed by WES in the study group but found by a targeted sequencing is the c.1746-20C>G variant. It involves a substitution of cytosine to guanine in intron 13, 20 nucleotides upstream of the first coding nucleotide of exon 14. This variant was not reported in the earlier WES study, as it is located deeper in the intron than typical splice site mutations, and its frequency is fairly high (0.4% in the NFE according to the gnomAD database). Although there are conflicting reports regarding its pathogenicity in the ClinVar database, it has been widely reported in European LGMD patients, although only in individual cases (Stehlikova et al., 2014; Peric et al., 2019; Topf et al., 2020). This study demonstrates the relative prevalence of this variant among apparently heterozygous LGMDR1 patients. In the studied group, it was present in eight patients in a compound heterozygous state, co-segregating with the disease in the family. Considering its frequency in the general population, it is intriguing that we found it only together with missense mutations or in-frame deletions but not with the most common del550A mutation. This finding, although of potential significance, should be interpreted carefully due to the small size of the group.

Another unexpected finding is that the c.1746-20C>G variant seems to be in a linkage disequilibrium with two deep intronic variants (c.310-893C>T, c.1782+223A>G) and a rare variant in putative regulatory region g.42406949C>G. To the best of our knowledge, this work is the first report of this linkage disequilibrium. The pathogenicity of either of the three linked variants (rather than the c.1746-20C>G variant *per se*) cannot be excluded.

It is also puzzling that no subgroup B patient was homozygous for the c.1746-20C>G variant. It could be explained by the small number of patients. However, we cannot exclude that homozygous individuals are asymptomatic or paucisymptomatic, especially considering the relatively mild phenotype of the c.1746-20C>G compound heterozygotes. From the pedigree analysis, we can infer that the deceased father of patient A6 could have been such an asymptomatic homozygote because two of the children have not inherited the g.42347848C>T variant, which is present in the remaining three children. It can be also possible that the pathogenicity of the c.1746-20C>G variant depends on the presence of other rare variants, i.e., located in the regulatory regions. It is of interest that seven out of nine patients with the c.1746-20C>G variant carried, besides the linked g.42406949C>G variant, another low-frequency variant in a putative regulatory region.

Western blotting revealed an absent or very weak calpain-3 band in compound heterozygotes with the c.1746-20C>G variant. This is an unexpected finding, especially considering that the second mutation is a non-null mutation. In such cases, one could expect at least partially preserved calpain-3 synthesis, also in line with the less severe course of the disease in these patients. Loss of calpain-3 protein in non-null mutations has been already described in the literature. In the paper by Nascimbeni et al. (2010) for five different missense mutations, each in combination with the c.1746-20C>G variant, the amount of calpain-3 detected with Western blot varied between 0 and 20% of the normal expression. Also for the in-frame c.598_612del15 deletion, calpain-3 protein deficiency (Cerino et al., 2020a) or even total absence was reported (Hermanova et al., 2006). The influence of the c.1746-20C>G variant on calpain-3 mRNA transcription has been studied by several authors, with contrasting results. Some deemed this variant non-pathogenic (Krahn et al., 2007; Stehlikova et al., 2007) because no abnormality was found on the mRNA or cDNA level. On the contrary, another group of authors, with the aid of splice-site predictor program, and splicing-specific primers, demonstrated that the c.1746-20C>G variant creates cryptic splice sites and leads to a partial exonization of intron 13. The mis-spliced transcripts are pathogenic, but at the same time, alternative splicing also leads to insertion of the entire intron 13, and the latter transcript seems non-pathogenic (Nascimbeni et al., 2010). The splicing abnormalities could lead either to lowered expression or a shorter half-life of calpain-3, which could account for the barely detectable protein level. Another yet unlikely explanation of this finding is that the antibody used for Western blotting does not recognize the extended protein with the entire intron 13 exonized.

### The Remaining Intronic Variants

Variant c.1194-9A>G is a known pathogenic variant, possibly affecting the splice site. Due to the data filtering algorithm, it was not detected during a previous WES study, which covered splicing sites up to -6 nucleotide.

Other intronic variants found are deep intronic. Their significance is unknown; however, deep intronic variants have been described in LGMDR1 (Blazquez et al., 2013; Hu et al., 2019) and are supposed to be pathogenic by the introduction of cryptic exons. In our only patient with two deep intronic compound heterozygous mutations (B2), Western blot revealed a high level of calpain-3, which points to their non-pathogenic character. However, the preserved synthesis of calpain-3 does not exclude loss of its enzymatic function (Fanin et al., 2007). Unfortunately, RNA studies of our patients were not possible as only frozen biopsy specimens were available.

### 3′UTR Mutation

Mutation in the 3′UTR region was already described in the *CAPN3* gene (Topf et al., 2020), and in proband A7, this is a putative pathogenic variant. The coexisting rare RYR1 variant found does not correspond neither with clinical presentation nor autosomal recessive inheritance. The patient has typical clinical LGMDR1 course with onset in adolescence. The 3’UTR mutations were described in other diseases as causing mRNA instability (Riachi et al., 2019; Bachetti et al., 2021; Cini et al., 2021).

### Large Deletion

Large deletion comprising exons 2–8 is present in proband A3 and C1. These are non-consanguineous female patients, which indicates possible prevalence of this mutation in Poland. This deletion is difficult to see by the WES method due to encompassing large number of exons. The same large deletion has been already reported in other populations (Krahn et al., 2007; Todorova et al., 2007). We suggest the necessity of targeted testing of undiagnosed LGMD patients for this deletion.

### Variants in Putative *CAPN3* Enhancers

Mutations in regulatory sequences are known to be potentially pathogenic. However, identification of functional non-coding mutations remains very challenging, and only a few have been already associated with various diseases (Epstein, 2009; Diederichs et al., 2016; Rojano et al., 2019). No such variants were described in LGMD, including LGMDR1. Although a rare g.42341662C>G variant was identified in this study in three families, and some rare variants identified in other patients were located in a relative vicinity (within 10 kbp), none of them can be proven to be pathogenic after segregation analysis. Another, g.42406949C>G, variant was found only in cases in which coding sequence mutation and c.1746-20C>G variant are the identified cause of the disease.

The pathogenicity or disease-modifying influence of identified regulatory sequence variations cannot be excluded, but pinpointing such causative variants requires studies on larger cohorts of patients with only one mutation in the *CAPN3* coding sequence.

### Previously Undetected Exonic Mutations

The targeted whole *CAPN3* gene sequencing revealed two exonic mutations that should have been detected by earlier screening: a point mutation in patient B1 was not reported in WES results, and a homozygous c.550delA mutation in patient C3 was originally diagnosed as heterozygous with Sanger sequencing. Re-assessing the WES data confirmed that the first mutation was present, but not reported previously due to low coverage in this part of the *CAPN3* sequence (<10×), which misled the variant calling program. The source of the second obvious error is unknown. The Sanger sequencing was performed more than 20 years ago outside our laboratory, and we had no access to the original data.

### Patients With “Double Trouble”

For probands A1, A2, and B6, we previously found mutations in myopathy-related genes in *FKRP*, *LMNA*, and *CCDC78*, respectively. As proband A1 has a very early onset, Duchenne-like course, clinical diagnosis of LGMDR5 is more plausible, although the modifying effect of *CAPN3* mutations is possible.

The proband A2 carrying a novel *LMNA* mutation presents both LGMD phenotype (which can be caused either by *LMNA* or *CAPN3* mutation) and cardiomyopathy (not characteristic for LGMDR1). The autosomal recessive inheritance of LGMD in this family supports the possibility that the two *CAPN3* rare variants are causal of LGMD in this case. Cardiomyopathy can be related to the *LMNA* mutation. Unfortunately, the family of the patient was not available for DNA testing. In the same patient, we also found a considerable number of rare variants of uncertain significance in other LGMD-related genes. Despite this apparent mutational burden, his disease progressed slowly, and at the age of 77, he was still ambulant.

Proband B2, besides having two deep intronic variants in the *CAPN3* gene, has also a novel mutation in the *CCDC78* gene, related to centronuclear myopathy. The patient reported generalized muscular weakness since childhood and developed significant scoliosis during school years; however, despite such an early onset, she remained ambulatory until the fifth decade of life. Increased number of centrally located nuclei within muscle fibers was reported in the muscle biopsy, together with other unspecific myopathic changes. The patient presents an atypical phenotype with some features of centronuclear myopathy as well as LGMD.

### Asymptomatic Patient With Compound Heterozygous Mutation

The brother of proband B1, carrying the same variants as the proband, is clinically asymptomatic, although he was toe walking as a child. His CK level is unknown. It is possible that he is still in the presymptomatic phase at the age of 26. Alternatively, other disease-modifying genes can play a role, among them, a rare variant in RYR1 found in the proband, but absent in her brother (data not shown). Interestingly, the p.Gly445Arg mutation found in both siblings has been reported in cases of autosomal dominant calpainopathy, with possible late-onset and variable symptoms (Cerino et al., 2020b; Gonzalez-Mera et al., 2021). In light of this information, the role of c.1746-20C>G and the linked variants in this family is uncertain.

### Unsolved Cases

After the targeted NGS of the whole *CAPN3* gene, we were able to diagnose or suspect LGMDR1 in most patients with only one *CAPN3* mutation detected previously. The only patient remaining undiagnosed in this subgroup is patient A13. The patient harbors a missense p.Asp753Asn mutation and two rare variants in putative regulatory regions. This patient also, as the only one from the WES-CAPN3het group, has preserved *CAPN3* expression in the muscle sample. It is, therefore, possible that the undetected causative mutations lie in other genes. Alternatively, this case could represent an autosomal dominant calpainopathy. Dominantly inherited forms of the disease are characterized by intrafamilial variability, and clinically asymptomatic mutation carriers are sometimes found (Vissing et al., 2016, 2020; Martinez-Thompson et al., 2018; Spinazzi et al., 2021). However, to date, the p.Asp753Asn mutation has not been associated with dominantly inherited calpainopathy. The influence of regulatory sequence variants can also play a role in this case.

In the WES-neg group, we were not able to confirm unambiguously the LGMDR1 diagnosis in majority of the patients. In three patients (B9, B10, and B7), there are genetic or clinical findings that can point to another type of LGMD.

Patients B9 and B10 carry rare variants in *COL6A3* (two in each patient) for which conflicting interpretations of pathogenicity exist. We cannot exclude that a single *COL6A3* variant, or a combination of both, is pathogenic in these cases. Clinical reassessment and further diagnostic procedures are needed to confirm LGMDD5 diagnosis for these patients. The novel *CACNA1S* mutation in patient B10 can also lead to a muscle disease, although the LGMD phenotype would be unusual for this gene (Flucher, 2020).

Patient B7 is the only one from the study group in whom no single rare variant in any LGMD-related gene (or putative regulatory sequence) was found. Given the Miyoshi phenotype, targeted genetic testing for deep intronic *DYSF* mutation is warranted in his case.

In the remaining patients, we have no clues pointing to a specific LGMD type. However, prevalence of rare intronic and putative regulatory sequence variants in this group is high, which indicates the possible abnormalities of *CAPN3* expression or function.

## Limitations

We are aware of some limitations of this work: (1) The variants were not confirmed by Sanger sequencing. However, targeted NGS confirmed all *CAPN3* variants previously found in the original study. (2) Segregation analysis of variants could not be performed for all families, in most cases, due to the unavailability of family members. (3) The putative regulatory sequences investigated here were chosen from the GeneHancer database of genome-wide enhancer-to-gene and promoter-to-gene associations, and their role in the regulation of *CAPN3* expression has not been experimentally validated. (4) Western blot was done only when at least one *CAPN3* mutation was detected in a given patient. We decided not to perform Western blot in all patients in order to save the biopsy tissue for planned future analysis of other proteins. (5) Analysis of transcripts was not performed We had previously tried to do mRNA analyses from frozen muscle biopsy samples of other LGMD patients, but those efforts were unsuccessful due to a high degree of RNA degradation.

## Conclusion

The presented results indicate that rare intronic variants of *CAPN3* are fairly common in Polish patients with a clinical diagnosis of LGMDR. Most likely, these variants are causative or co-causative, and further *in vitro* functional analyses are necessary to elucidate their pathogenicity. The high prevalence of previously undetected mutations indicates that routine testing for at least non-coding variants in the *CAPN3* locus or preferably WGS should be performed in patients for whom only one heterozygous mutation was detected. The c.1746-20C>G variant seems to be common in Poland and seems to correlate with a relatively mild phenotype. Large deletions, especially the 2–8 exon deletion, should be also taken into account during genetic testing as they cannot be detected routinely with WES.

## Data Availability Statement

The data presented in the study are deposited in the European Variation Archive repository, project accession number PRJEB46068, analyses accession number ERZ2774435.

## Ethics Statement

The studies involving human participants were reviewed and approved by the Ethics Committee of the Medical University of Warsaw, Poland. Written informed consent to participate in this study was provided by the participants’ legal guardian/next of kin.

## Author Contributions

AM and JPF designed the study and analyzed the putative influence of variants on phenotype. AK-P supervised the clinical part of the research. MR supervised the biochemical part of the research. AM, AK, and AK-P recruited the patients and conducted the clinical phenotyping. JPF performed the selection of sequences for NGS and analysis of the prefiltered datasets. MT performed the Western blotting. AM, JPF, MR, and AK-P drafted and edited the manuscript. All authors reviewed and commented on the manuscript, and read and approved the final manuscript.

## Conflict of Interest

The authors declare that the research was conducted in the absence of any commercial or financial relationships that could be construed as a potential conflict of interest.

## Publisher’s Note

All claims expressed in this article are solely those of the authors and do not necessarily represent those of their affiliated organizations, or those of the publisher, the editors and the reviewers. Any product that may be evaluated in this article, or claim that may be made by its manufacturer, is not guaranteed or endorsed by the publisher.

## Abbreviations

BWA: Burrows–Wheeler alignment (software)
CK: serum creatine kinase
ECL: enhanced chemiluminescence
ExAC: The Exome Aggregation Consortium (database)
GAPDH: glyceraldehyde 3-phosphate dehydrogenase
GATK: Genome analysis toolkit (software)
HGMD: Human Gene Mutation Database
HPO: The Human Phenotype Ontology (database)
indel: small insertion/deletion
LGMD: limb–girdle muscular dystrophy
MLPA: multiplex ligation-dependent probe amplification
NFE: Non-Finnish European population
MAF: minor allele frequency
NGS: next-generation sequencing
Sang-CAPN3het: subgroup of patients, in whom *CAPN3* heterozygous mutations were found with Sanger sequencing only
SNV: single-nucleotide variation
VUS: variant of unknown significance
WES: whole-exome sequencing
WES-CAPN3het: subgroup of patients with only one *CAPN3* mutation detected with WES
WES-neg: subgroup of patients with no causative mutation found with WES
WGS: whole-genome sequencing.
