# Supplementary figures and images for: Targeted Next-Generation Sequencing Reveals Mutations in Non-coding Regions and Potential Regulatory Sequences of Calpain-3 Gene in Polish Limb–Girdle Muscular Dystrophy Patients
Source: Front Neurosci. 2021 Oct 14;15:692482. doi: 10.3389/fnins.2021.692482 (PMC8551377; doi:10.3389/fnins.2021.692482)

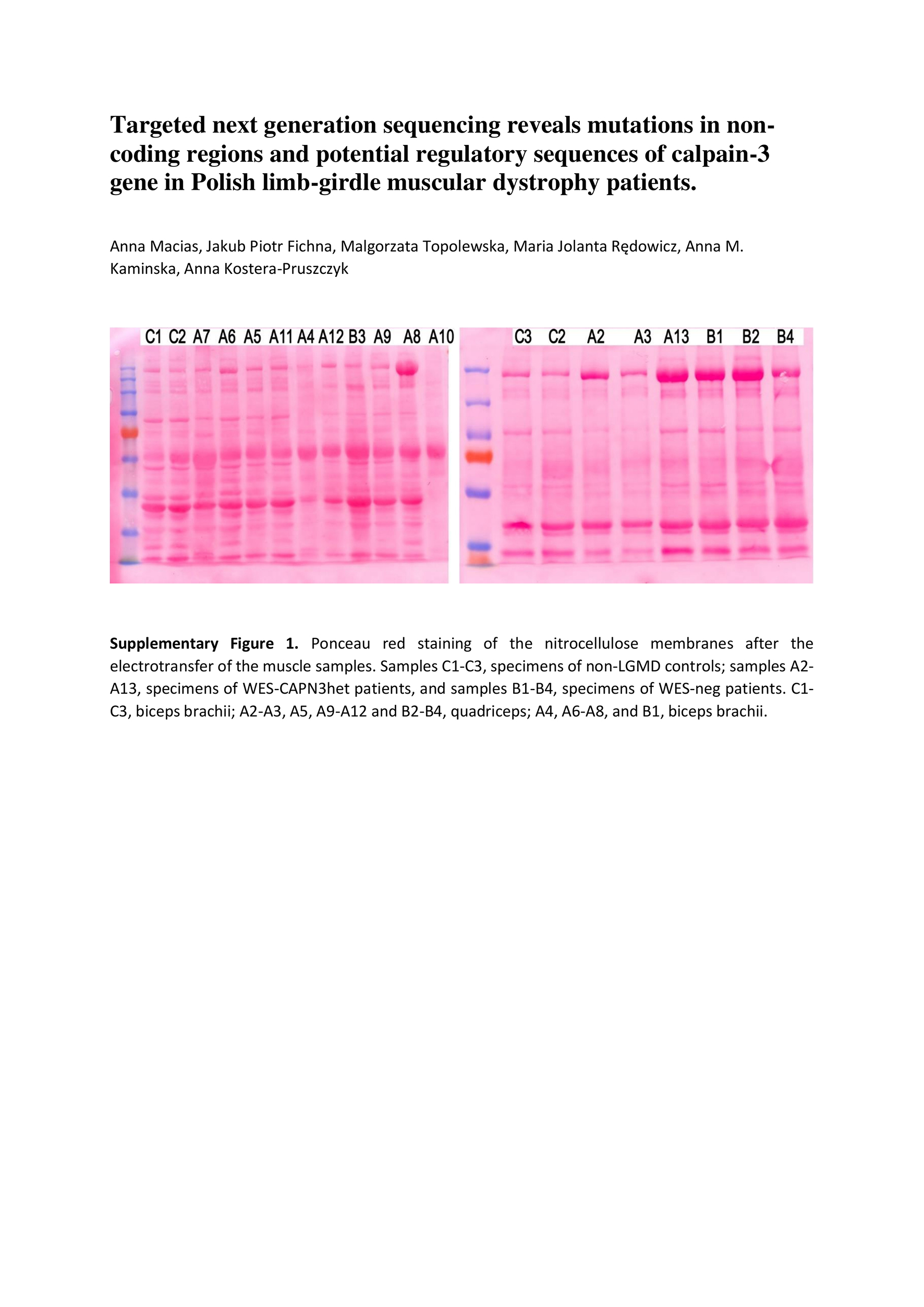

Supplement: Supplementary file 1 [file Image_1.JPEG]
