# Supplementary material for: Targeted Next-Generation Sequencing Reveals Mutations in Non-coding Regions and Potential Regulatory Sequences of Calpain-3 Gene in Polish Limb–Girdle Muscular Dystrophy Patients
Source: Front Neurosci. 2021 Oct 14;15:692482. doi: 10.3389/fnins.2021.692482 (PMC8551377; doi:10.3389/fnins.2021.692482)
Supplement: Supplementary file 2 [file Table_1.docx]

| **Patient** | **Number/symbol in the**  **original cohort** | **Mutations in other LGMD genes** | | |
| --- | --- | --- | --- | --- |
|  |  | **known pathogenic or**  **frameshift/stop variants** | **novel variants** | **other genes with rare variants^#^** |
| **A1** | CM | *FKRP* p.Leu93Pro/ p.Arg270Cys |  | *DMD, NEB, SYNE1 x2, CCDC78, TTN x4* |
| **A2** | 19 | *COL6A3* p.Glu1386Lys^##^  *DYSF* p.Arg1022Gln + p.Arg1331Leu | *LMNA* p.Gly523Arg  *PLEC1* p.Phe4379Leu  *RYR1* p.Ser3163Pro + p.Ser2721Phe | *PLEC x2, HSPG2, SYNE1, MYH3, LMOD3,* |
| **A3** | 448a | *TTN* Leu3558Thrfs |  |  |
| **A4** | 179 |  |  | *COL6A2, DNM2, BVES, TTN x4* |
| **A5** | 214 | *COL6A3* p.Glu1386Lys^##^ |  | *POMT2, COL12A1,*  *TTN x2* |
| **A6** | 191 | *FKRP* p.Ala114Gly |  | *TTN x3* |
| **A7** | 170 | *SGCA* p.Gly193fs |  | *RYR1, CACNA1S, LDB3/ZASP* |
| **A8** | 250a |  | *SGCD* p.Leu200Arg | *HSPG2, TTN* |
| **A9** | 160a | *DES* p.Ala213Val |  | *BAG3, NEB x2, TTN x2, CACNA1S* |
| **A10** | 752 | *POMT2* p.Tyr354fs |  | *FLNC x5, NEB, HSPG2, SYNE2, TTN* |
| **A11** | 130a |  | *PLEC* p.Ala1082Val | *PLEC, SYNE1 x2, SYNE2, CACNA1S, TTN* |
| **A12** | 658 |  |  | *MYPN, TARDBP, TTN x2* |
| **A13** | - |  |  |  |
| **B1** | 9 |  | *RYR1* p.Asp4111Ile | *NEB, MYH7, FLNC, TTNx2* |
| **B2** | 667 |  | *CCDC78* p.Arg103Gln | *SYNE1, NEB x2, MTM, TTN x2* |
| **B3** | 128a | *RYR1* p.Arg3347His |  | *RYR1, COL6A3* |
| **B4** | 14 |  |  | *FLNC x2, TTN x2* |
| **B5** | 592 |  |  | *BAG3, TMEM43, TTN x3, HSPG2* |
| **B6** | 195 |  |  | *DNM2, TRIM32, POMGNT1, FLNC, NEB* |
| **B7** | 17 | *POLG* p.Ala467Thr |  | *DMD, PLEC x2, LAMA2, ITGA7, MYH6, SYNE2, CACNA1S, NEB* |
| **B8** | 197 |  | *SCN4A* p.Gly760Glu | *COL6A3, ANO5, NEB, COL12A1,*  *MYH3, SYNE1, TTN x2* |
| **B9** | 7 | *COL6A3* p.Arg2142* / p.Lys2483Glu |  | *FLNC* |
| **B10** | 901 | *COL6A3* p.Glu1386Lys^##^ | *CACNA1S* p.Thr349Ser | *COL6A3, NEB, TTN* |

^#^ - for better table readability, only gene names are listed

^##^ - this variant pathogenicity is questionable (National Center for Biotechnology Information. ClinVar; [VCV000128819.6], https://www.ncbi.nlm.nih.gov/clinvar/variation/VCV000128819.6 (accessed June 8, 2021) Global Variome shared LOVD. COL6A3 (collagen, type VI, alpha 3) https://databases.lovd.nl/shared/view/COL6A3?search_VariantOnGenome%2FDBID=%3D%22COL6A3_000005%222021 (accessed June 8, 2021). Besides, its gnomAD allele frequency is 0.01085, which indicates that it is too common to cause a rare dominantly inherited disease;
