# Supplementary material for: Targeted Next-Generation Sequencing Reveals Mutations in Non-coding Regions and Potential Regulatory Sequences of Calpain-3 Gene in Polish Limb–Girdle Muscular Dystrophy Patients
Source: Front Neurosci. 2021 Oct 14;15:692482. doi: 10.3389/fnins.2021.692482 (PMC8551377; doi:10.3389/fnins.2021.692482)
Supplement: Supplementary file 3 [file Table_2.DOCX]

| **Patient no.** | **Genotype** | **Age/Gender** | **Onset of overt clinical symptoms** | **Initial symptoms** | **Ambulation** | **Genera-lized muscle atrophy** | **Muscle hyper-trophy** | **Scapular winging** | **Ankle contractures** | **CK (x UNL)** | **Other \ Follow-up** |
| --- | --- | --- | --- | --- | --- | --- | --- | --- | --- | --- | --- |
| **A1** | p.Glu107Lys / c.309+7931C>T  *FKRP* p.Leu93Pro/ p.Arg270Cys | 10 F | early childhood | delayed motor milestones | lost in 1st decade | no | diffuse, also  calf muscles | no | no | 27 |  |
| **A2** | p.Glu217Lys / c.1746-20C>G | 76  M | adolescence | limb-girdle weakness | waddling gait | yes | no | no | no | 7 | exacerbation with myalgia after URTI at 73; cardiac arrhythmia; asymmetry |
| **A3** | p.Gly161Arg / c.1746-20C>G | 56  F | adulthood | limb-girdle weakness | ambulation with assistance | yes | prominent calves | no | no | 2.5 |  |
| **A4** | p.Gly234Arg / c.1746-20C>G | 57  F | adolescence | limb-girdle weakness | ambulation with assistance | yes | no | no | no | 6 |  |
| **A5** | c.598-612delGTTCTGGAGTGCTCT/ c.1746-20C>G | 41  F | adulthood | limb-girdle weakness | waddling gait | yes | no | no | no | 22 | follow-up: at 54 loss of ambulation |
| **A6** | c.598-612delGTTCTGGAGTGCTCT/ c.1746-20C>G | 35  M | adulthood | muscle atrophy | waddling gait | yes | calf muscles | yes | no | 1.5 | asymmetrical upper limb onset; shoulder girdle muscle atrophy; |
| **A7** | c.598-612delGTTCTGGAGTGCTCT/ c.1746-20C>G | 42  M | adulthood | limb-girdle weakness | preserved | no | calf muscles | no | no | 4 | fatigability and muscle cramps |
| **A8** | c.598-612delGTTCTGGAGTGCTCT/ c.1746-20C>G | 44  M | adulthood | limb-girdle weakness | preserved | no | yes | yes | no | 12 | upper limb predominant phenotype |
| **A9** | p.Thr706Arg / c.1194-9A>G  + c.309+4853C>G | 16  F | adolescence | limb-girdle weakness | preserved | yes | calf muscles | no | no | 36 |  |
| **A10** | p.Arg533Ser/ c.1355-158C>A | 56  F | adulthood | limb-girdle weakness | preserved | no | no | no | no | 2.5 |  |
| **A11** | c.550delA / c.*32A>G | 15  M | adolescence | limb-girdle weakness | preserved | yes | no | yes | no | 27 |  |
| **A12** | c.1722delC / del exons 2-8 (g.42364582_42395592del) | 31  F | adolescence | limb-girdle weakness | waddling gait | yes | no | yes | no | 3 |  |
| **A13** | p.Asp753Asn / - | 30  F | adulthood | myalgia, fatigability | preserved | no | no | no | no | 6 | Pseudometabolic phenotype |
| **B1** | c.1746-20C>G / p.Gly445Arg | 14  F | early childhood | toe walking | preserved | no | prominent calves | no | tempo-rary | 9 | at 26 asymptomatic; ankle contractures resolved |
| **B2** | c.309+1668A>T / c.309+5112C>T  *CCDC78* p.Arg103Gln | 48 F | childhood | limb-girdle weakness | preserved | no | no | no | no | 15 | myalgia; slow progression initially, acceleration in adulthood at  … ambulatory with assistance |
| **B3** | c.1746-20C>G / - | 10 F | childhood | toe walking | waddling gait | severe | no | no | no | 81 | fast progression; ambulation loss at 1136 |
| **B4** | c.310-8109A>G / - | 8 F | - | - | normal | no | prominent calves | no | no | 80 | asymptomatic hyper-CKmia |
| **B5** | - / - (MAF 1-3%: c.1194-856T>C;  c.1524+81C>T) | 36 F | adolescence | limb-girdle weakness | preserved | yes | no | no | no | 1.4 | myalgia and muscle cramps; asymmetry |
| **B6** | - / - | 17  M | adolescence | muscle atrophy | preserved | severe | no | no | yes | 47 |  |
| **B7** | - / - | 34  M | early adulthood | muscle atrophy | waddling gait | calf muscles | no | no | no | 24 | predominantly distal leg weakness |
| **B8** | - /- (MAF 1-3%: c.1354+543C>T) | 9  F | - | - | normal | no | no | no | no | 20 | asymptomatic hyper-CKmia |
| **B9** | *COL6A3* p.Arg2142* / p.Lys2483Glu | 14  F | childhood | limb-girdle weakness | preserved | no | no | no | no | 7 |  |
| **B10** | *COL6A3* p.Glu1386Lys  *CACNA1S* p.Thr349Ser | 41  M | adulthood | limb-girdle weakness | waddling gait, knee hyper-extension | yes | prominent calves | yes | no | 1.9 | also mild distal upper limb weakness |
| **C1** | c.550delA / del exons 2-8 (g.42364582_42395592del) | 27 F | childhood | limb-girdle weakness | with assistance | no data^#^ | no | no data^#^ | no data^#^ | UK |  |
| **C2** | c.550delA / p.Lys254del | 12 F | childhood | limb-girdle weakness | waddling gait | no data^#^ | no | no data^#^ | no data^#^ | UK | follow-up: loss of ambulation in 3^rd^ decade |
| **C3** | c.550delA / c.550delA | 12 M | childhood | toe walking | waddling gait | no data^#^ | no | no data^#^ | yes | UK | follow-up: loss of ambulation in 3^rd^ decade |
| **C4** | c.550delA /  c.598-612delGTTCTGGAGTGCTCT | 23 M | childhood | toe walking | wheelchair | no data^#^ | prominent calves | no | yes | UK | follow-up: loss of ambulation in 3^rd^ decade |

^#^ - patients in group C were included in the study many years after the initial diagnosis; detailed clinical description from the time of diagnosis were not available, hence lack of some clinical data;

Abbreviations: F: female, M:male, CK – creatine kinase; UNL: upper normal limit; UK: unknown; URTI: upper respiratory tract infection;
